# Curing of Poly(styrene-co-methyl methacrylate-co-2-hydroxyethyl methacrylate) Terpolymers in the Presence of Amino Compounds of Different Structures

**DOI:** 10.3390/polym15092187

**Published:** 2023-05-04

**Authors:** Tatyana A. Pugacheva, Vladimir G. Kurbatov, Evgeniy V. Vaganov, Georgiy V. Malkov, Ksenya A. Tarusina, Vlada M. Organ, Elena V. Mitrofanova, Alexander E. Tarasov, Elmira R. Badamshina

**Affiliations:** 1 Federal Research Center of Problems of Chemical Physics and Medicinal Chemistry, Academician Semenov Avenue 1, Chernogolovka 142432, Russia; sinichka.71@yandex.ru (T.A.P.); wag28332@yandex.ru (E.V.V.); gmalkov@mail.ru (G.V.M.); ktarusina@bk.ru (K.A.T.); atarasov@icp.ac.ru (A.E.T.); badamsh@icp.ac.ru (E.R.B.); 2 Department of Chemical Technology of Organic Coatings, Yaroslavl State Technical University, Moscow Avenue 88, Yaroslavl 150023, Russia; vlada.organ@mail.ru (V.M.O.); mitrofanova_helena_15@mail.ru (E.V.M.)

**Keywords:** acrylic oligomers, crosslinking agent, catalyst, rheology, curing

## Abstract

The process of curing the acrylic oligomers for rapid thermal curing coatings in the presence of hexa(methoxymethyl)melamine (HMMM), tetra(butoxymethyl)glycoluril (TBMG), and tetra(methoxymethyl)glycoluril (TMMG) has been studied. When HMMM is used as a hardener, the content of hydroxyl groups in the terpolymer and also the crosslinking agent concentration have little effect on the initial cure rate. It has been established that during the curing of the TMMG composition, the amount of the network polymer and the initial curing rate decrease at short curing times only. It has also been revealed that the use of butoxy groups instead of methoxy groups as blocking agents leads both to a decrease in the initial cure rate and the gel fraction limiting value from 98 to 80%. When it comes to TBMG-containing compositions, a decrease in the part of hydroxyl groups in the copolymer leads to a significant fall in the initial curing rate and also in the gel fraction content. Regardless of the crosslinking agent used, an acceleration of the curing process is observed with an increase in the catalyst content in the compositions.

## 1. Introduction

Melamine-formaldehyde oligomers are widely used in paints and varnishes [1,2,3,4]. Generally, they can be obtained by polycondensation of melamine with formaldehyde and subsequent esterification with monoalcohols, such as methanol, n-butanol, and isobutanol [1,2].

Being influenced by the external factors such as temperature and catalyst, almost all melamine-formaldehyde oligomers are capable of forming three-dimensional products. Nevertheless, they have found no application in the paint and varnish industry as standalone film-forming agents [1,2]. It can be explained by the fact that coatings based on these oligomers are more brittle and possess low adhesion to the substrate. Therefore, they are generally used together with other types of film-forming agents, primarily with alkyd, polyester, and epoxy resins, also with unmodified oligoesters and unsaturated oligoesters containing functional groups such as OH— or COOH—, capable of relatively easily reacting with methylol and alkoxymethyl groups. Melamine-formaldehyde oligomers provide hardness, gloss, and color stability to the coatings under the influence of UV rays, as well as resistance to water, gasoline, mineral oils, and mild alkaline detergents.

Systems based on polyesters and melamine-formaldehyde oligomers are widespread in industry. They make it possible to obtain coatings used for painting rolled metal [5,6,7,8]. The combination of polyester with hexa(methoxymethyl)melamine (HMMM) results in higher elasticity, adhesion, and protective properties of coatings, as well as shorter cure time at fairly high temperatures.

Amino-formaldehyde oligomers such as tetra(methoxymethyl)glycoluril (TMMG) have also found application as crosslinkers in the production of coatings based on powdered film forming systems, e.g., for epoxy and polyester ones [9,10,11,12].

Compositions containing amino-formaldehyde oligomers can also be used in microelectronics, apart from paint coatings. For example, systems containing amino-formaldehyde oligomers as hardeners are used to obtain cross-linked negative photoresist films based on p-hydroxystyrenes, their derivatives, and some other oligomers [13,14,15,16,17]. Moreover, in all these cases, the curing process is not given enough attention.

Another application for compositions containing amino-formaldehyde oligomers is the preparation of anti-reflective coatings for the 193 nm photolithography [18,19,20]. It is noteworthy that all these articles listed above cover no curing process aspects as well.

Obtaining anti-reflective coatings is an important part of the photolithography in the manufacture of large integrated circuits; therefore, rather high requirements are imposed on them. In particular, it is necessary to obtain coatings with a relatively small thickness (~100 nm); meanwhile, their curing should take place within 90 s until high conversion values, so that the coating could not swell in the photoresist components after curing. To meet these requirements, it is important to select reasonably the oligomer structure and the hydroxyl group content in it, the chemical structure of the crosslinking agent, and the catalyst, as well as their amount in the composition.

The aim of the present work is to study the curing process of compositions based on acrylic terpolymers with various 2-hydroxyethyl methacrylate (HEMA) content in the presence of cross-linking agents of various structures.

## 2. Materials and Methods

Styrene-acrylic oligomers were used as research objects, their compositions are given in Table 1.

As the crosslinking agents, this study used hexa(methoxymethyl)melamine (HMMM) (CYMEL^®^ 303 LF, ≥98%, Allnex, Hamburg, Germany), tetrakis(methoxymethyl)glycoluril (TMMG, ≥98%, Tokyo chemical industry, Tokyo, Japan), and tetrakis(butoxymethyl)glycoluril (TBMG) (CYMEL^®^ 1170, ≥98%, Allnex, Hamburg, Germany). The crosslinking agent content ranged from 4.3 to 17 wt.% of the copolymer weight. The curing catalyst used was p-Toluenesulfonic acid (PTSA) (97%, OOO Chemical Line, Saint Petersburg, Russia). The curing catalyst content ranged from 2.5 to 10 wt.% of the crosslinking agent. To prepare the composition, the copolymer was dissolved in a selected solvent; then, a crosslinking agent and a curing catalyst were added. The compositions were cured in the desiccator at 215 °C for 120 s. The rheological properties of copolymer solutions were studied using a «plate–plate» measuring system on an MCR 702 TwinDrive rheometer (Anton Paar, Graz, Austria). The measurements were carried out under the controlled shear stress within the 0 to 15 Pa range. The rheology was investigated at 25 °C.

These properties were studied for the solutions of terpolymers with a concentration of 20 wt.% in methoxypropanol (MOP, 99.5%, Sigma Aldrich, St. Louis, MO, USA), methoxypropyl acetate (MPA, ≥96%, Sigma Aldrich, Missouri, USA), and tetrahydrofuran (THF, 99.5%, Sigma Aldrich, St. Louis, MO, USA). The coatings were applied to glass substrates from terpolymer solutions (copolymer content in the solution of 20 wt.%) using a slot applicator with a 100 µm slot.

The content of the mesh polymer in the epoxy composition was determined by sol-gel analysis in a continuously operating Soxhlet extractor. The extraction solvent was acetone (99.0%, AO ECOS-1, Moscow, Russia); the extraction process continued for 6 h. After extraction, the plates were dried for 6 h at 105 ± 5 °C. The initial curing rate of the coatings was determined as the tangent slope in the linear section of the dependence of the gel fraction content on the curing time of the composition.

The swelling was determined by immersing a pre-weighted glass plate with a cured coating in MPA, which is most commonly used as a solvent for photoresists at an operating wavelength of 193 nm. The coatings were immersed in MPA for 12 h. After that, the plates were taken out of the solvent, dried with filter paper, and kept in the draft chamber at room temperature for 30 min. After that, the coatings were weighed, and the swelling degree was determined.

Fourier-transform infrared spectroscopy (FTIR) analysis of coatings was carried out in the range of 375–4000 cm^−1^ (2 cm^−1^ resolution) using BRUKER ALPHA (Bruker Optics, Billerica, MA, USA). The number of scans was 32.

## 3. Results

The rheological properties of the copolymer solutions will determine the methods of applying coating, as well as help in determining the optimal conditions of applying coating that ensure obtaining films of the required thickness. MOP and MPA were selected as solvents because they are the most common in manufacturing compositions for anti-reflective coatings. THF is used as a solvent because it is highly soluble and is used for the synthesis of terpolymers, so the study also evaluated its use as a solvent for obtaining compositions.

**Figure 1:**
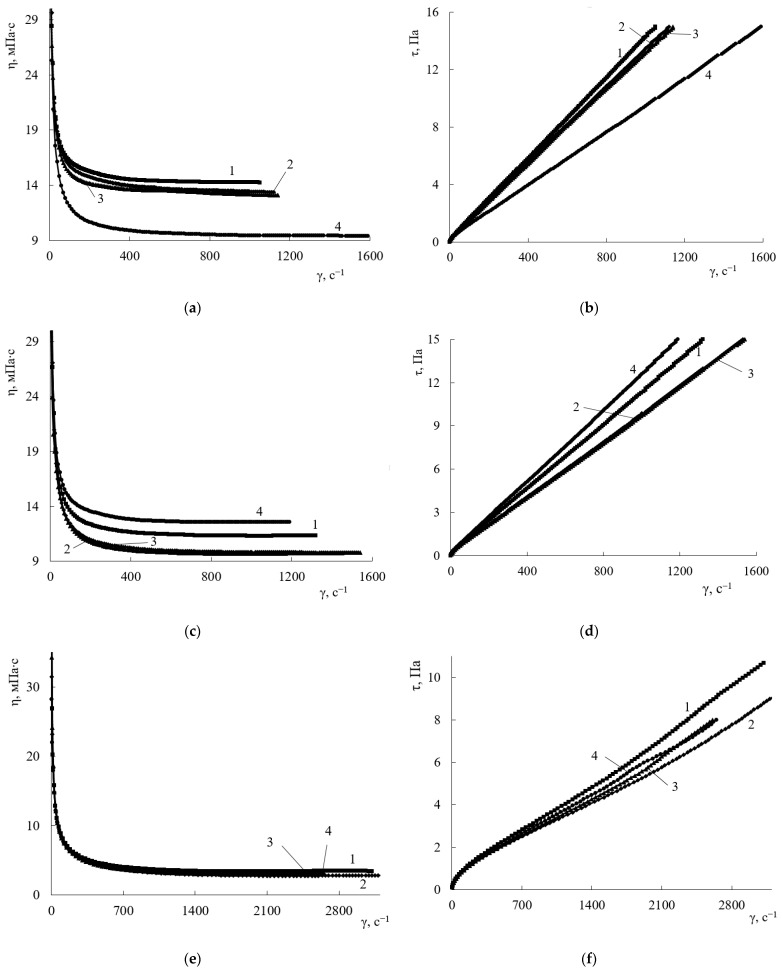
Viscosity (**a**,**c**,**e**) and flow (**b**,**d**,**f**) curves for solutions of MMA-ST-HEMA terpolymers in MOP (**a**,**b**), MPA (**c**,**d**), THF (**e**,**f**) HEMA content, mol.%: 1—45; 2—32; 3—25; 4—20.

When MOP is used as a solvent, the viscosity of the copolymer solution increases with increasing HEMA content in the copolymer (Figure 1a). This happens because, with increasing content of hydroxyl groups, the number of centers of strong intermolecular interaction also increases, leading to the structuring of polymer solutions (Table 1), which leads to an increase in viscosity. It should be noted that, with an increase in HEMA content from 20 to 25 mol.%, the viscosity of the terpolymer solution doubles. Further increase in the HEMA content increases the viscosity to a much lesser extent.

When using MPA as a solvent, the dependence of viscosity on the content of HEMA in the copolymer changes compared to the rheological dependences of solutions in MOP. The viscosity of the copolymer solution with the minimum HEMA content in MPA increases, while that of the other copolymers under study decreases, and the decrease in the solution viscosity is not proportional to the increase in the HEMA content in the copolymer. This is probably because, at high concentrations of HEMA, the solutions opalesce, which indicates incomplete dissolution of the copolymer and, consequently, its different concentrations in the dissolved state.

The content of HEMA links has little effect on the viscosity of the system when using THF as a solvent. Such dependences may be related to the lower viscosity of the solvent itself, as well as its lower affinity to the copolymers under study. This might be because THF is thermodynamically a poorer solvent for terpolymers compared to MOP and MPA.

For a terpolymer with high HEMA content (45 mol.%), the viscosity of terpolymer solutions increases in the series THF > MPA > MOP (Figure 1a,c,e, curve 1). Such rheological behavior of solutions can be caused by an increase in the polar component of the total solubility parameter of the selected solvents (Table 2). 

For terpolymers containing 20 mol.% HEMA, a higher viscosity is observed when MPA is used as a solvent, which is associated with a decrease in the content of hydroxyl groups and, consequently, a decrease in the polarity of the terpolymer (Figure 1a,c,e, curve 4).

It was shown that the terpolymer solutions in THF have the lowest viscosity. However, THF is a highly volatile solvent and will evaporate rapidly with boiling at the curing temperature of the compositions, resulting in a large number of coating defects. MPA-based solutions are also less viscous than MOP-based solutions. However, the solutions obtained using MPA remain opalescent, indicating incomplete dissolution of the polymer. Therefore, MOP was chosen as the solvent for obtaining compositions and coatings based on them. This solvent completely dissolves the polymer, and its boiling point is much higher compared to THF, which will reduce the number of defects in the coating formation.

**Figure 2:**
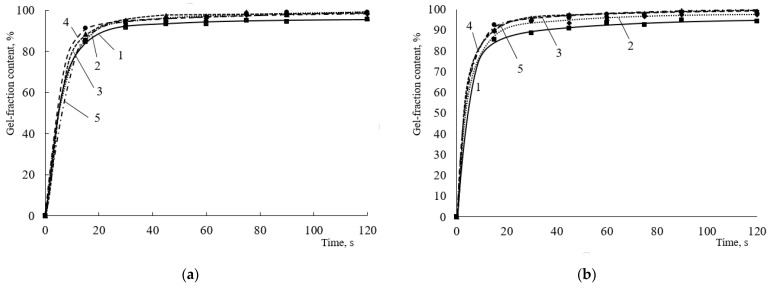

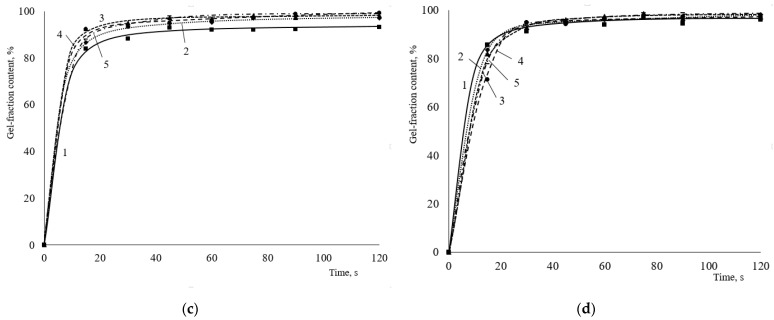
Dependence of the gel fraction content on the curing time of the compositions in the presence of HMMM (HEMA content in the terpolymer, mol.%: (**a**)—45; (**b**)—32; (**c**)—25; (**d**)—20. Catalyst content is 5 wt.% of mass of crosslinking agent) HMMM content, wt.%: 1—4.3; 2—6.5; 3—8.5; 4—12.8; 5—17.

The different structure of the terpolymer, in particular, the content of reactive hydroxyl groups, as well as the crosslinking agent content and type, can affect the curing process. When HMMM is used as a crosslinking agent, a high content of the crosslinking polymer is already observed in the initial moment, and it does not change significantly with increasing crosslinking agent content and curing time thereafter (Figure 2).

**Figure 3:**
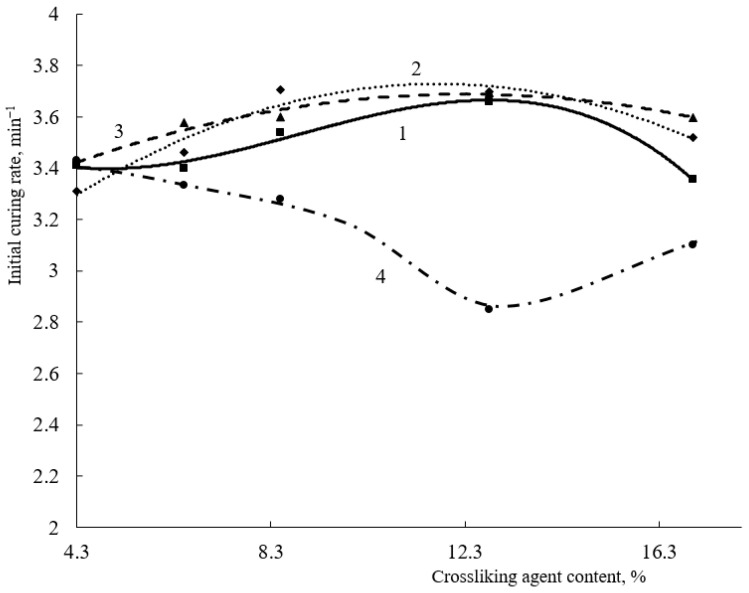
Dependence of the initial curing rate of compositions on the crosslinking agent content (HMMM was used as a crosslinking agent. Catalyst content is 5 wt.% of mass of crosslinking agent) HEMA content in terpolymer, mol.%: 1—45; 2—32; 3—25; 4—20.

The content of the gel fraction in all coatings after 15 s of curing is quite high and is at ~85% (Figure 2), and the initial curing rate ranges from 3.2 to 3.4 min^−1^ (Figure 3). When the content of HEMA links in the copolymer exceeds 20 mol.%, there is no significant increase in the gel fraction content as the amount of curing hardener increases. The maximum content of the gel fraction in the coating is observed at a hardener concentration of 6.5 wt.% (Figure 2). When the content of HEMA links in the copolymer is reduced to 20 mol.%, there are more significant differences in the curing process at the initial point in time. This is due to a decrease in the number of reactive groups in the terpolymer and, as a consequence, a decrease in the curing reaction rate. Notably, when 8.5% wt.% of the hardener was introduced into the composition, only the minimum content of the gel fraction was observed for the copolymer with 20 mol.% HEMA content. Probably, at this concentration of crosslinking agent in the presence of acid catalyst, the self-condensation reactions of HMMM become predominant. As a result, the acrylic oligomer will not be crosslinked and will dissolve in the acetone used to extract the coating. A further increase in the gel fraction content in the coatings obtained on the basis of terpolymer with 20 mol.% HEMA content is due to an increase in the proportion of self-condensation reactions of the crosslinking agent. These reactions also produce an insoluble polymer, which causes an increase in the gel fraction in the coating. According to [21], the self-condensation of HMMM mainly proceeds by two reactions:

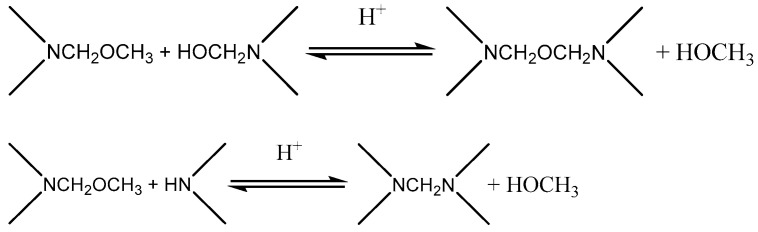



Free methylol groups in HMMM can form due to hydrolysis of alkoxyl groups during curing. The imino groups in HMMM can be formed as a result of formaldehyde detachment from methylol groups. In another study [22], the self-condensation is described by an even greater number of possible reactions that can occur in HMMM. The same study shows that increasing the catalyst content in the system increases the proportion of self-condensation reactions when HMMM is used as a crosslinking agent.

**Figure 4:**
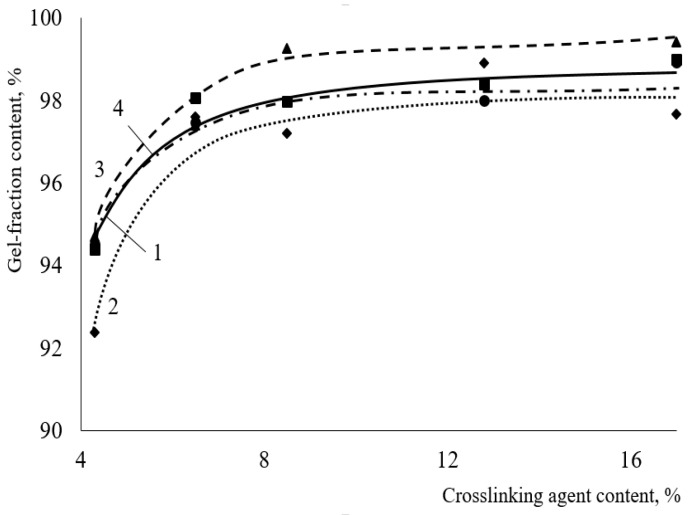
Dependence of the content of the gel fraction in the composition on the content of the crosslinking agent after two minutes of curing (HMMM was used as the crosslinking agent. Catalyst content is 5 wt.% of mass of crosslinking agent) HEMA content in terpolymer, mol.%: 1—45; 2—32; 3—25; 4—20.

At the end of curing (120 s), the amount of hydroxyl groups no longer affects the depth of curing of coatings. The change in the gel fraction content with increasing hardener concentration does not exceed 4% (Figure 4).

**Figure 5:**
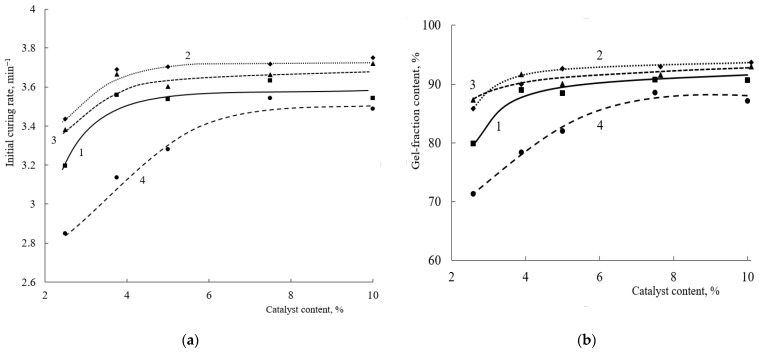
Dependence of initial curing rate of compositions (**a**) and amount of gel fraction (after 15 s of curing) (**b**) on curing catalyst content (Crosslinking agent concentration is 8.5 wt.%. HMMM was used as a crosslinking agent. HEMA content in the copolymer, mol.%: 1—45; 2—32; 3—25; 4—20).

In addition to the hardener content, the curing catalyst plays a significant role in such compositions. The greatest influence of the amount of catalyst, as well as of the crosslinking agent, is noticeable at short curing times (up to 15 s) (Figure 5). Increasing the catalyst content above 3.9 wt.% has no effect on either the mesh polymer content or the initial curing rate of the coatings. At the same time, a significant decrease in both the initial curing rate and the content of the mesh polymer is observed for the oligomer with a 20 mol.% HEMA content. For these oligomers, both the initial curing rate and the mesh polymer content increase with increasing catalyst concentration. The maximum gel fraction content for an oligomer with 20 mol% of HEMA links in the copolymer is observed at a catalyst concentration of 3.9 wt.%. Increasing the catalyst content will lead to an increase in the proportion of self-condensation reactions of the HMMM-based oligomer [10].

**Figure 6:**
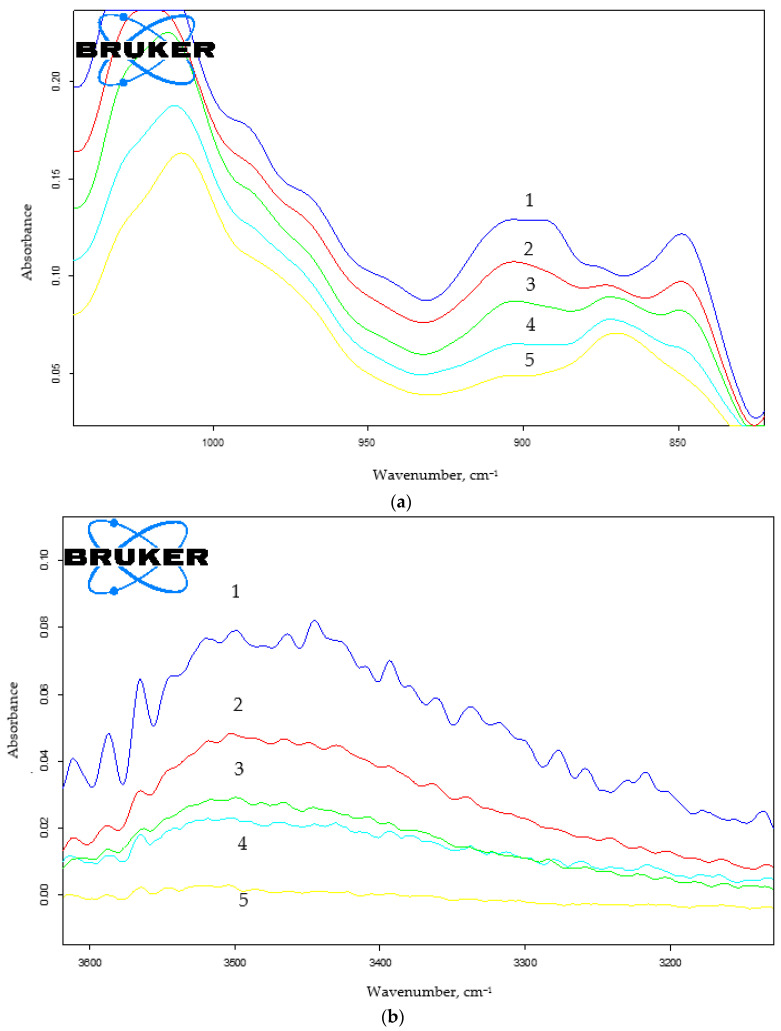
IR spectra of coatings based on terpolymer containing 45 mol.% of HEMA and HMMM as the curing agent. The area of varying the content of methoxyl (**a**) and hydroxyl (**b**) groups (PTSA was used as a catalyst in the amount of 5 wt.% of the curing agent. HMMM content, wt.%: 1—4.3; 2—6.5; 3—8.5; 4—12.8; 5—17).

The increase from 4.3 to 17 wt.% in the HMMM content led to the growth of the degree of curing for coatings made from the composition that contains oligomer of 45 mol.% of HEMA content (Figure 6 and Figure S1). It was evidenced by the decrease in the intensity of the absorption band at ~3500 cm^−1^ that corresponded to the oligomer hydroxyl group (Figure 6b). The decrease in the intensity of the absorption band at 905 cm^−1^, corresponding to the methoxyl group content in the system (Figure 6a), also confirmed the curing depth increase.

**Figure 7:**
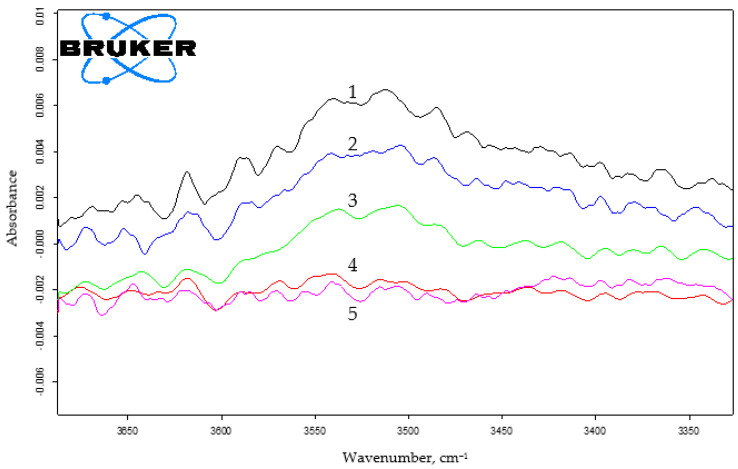
IR spectra of coatings based on terpolymer containing 20 mol.% of HEMA and HMMM as the curing agent. The area of varying the content of hydroxyl groups (PTSA was used as a catalyst in the amount of 5 wt.% of the curing agent. HMMM content, wt.%: 1—4.3; 2—6.5; 3—8.5; 4—12.8; 5—17).

A similar trend is observed for the oligomer of 20 mol.% of HEMA content (Figure 7 and Figure S2).

**Figure 8:**
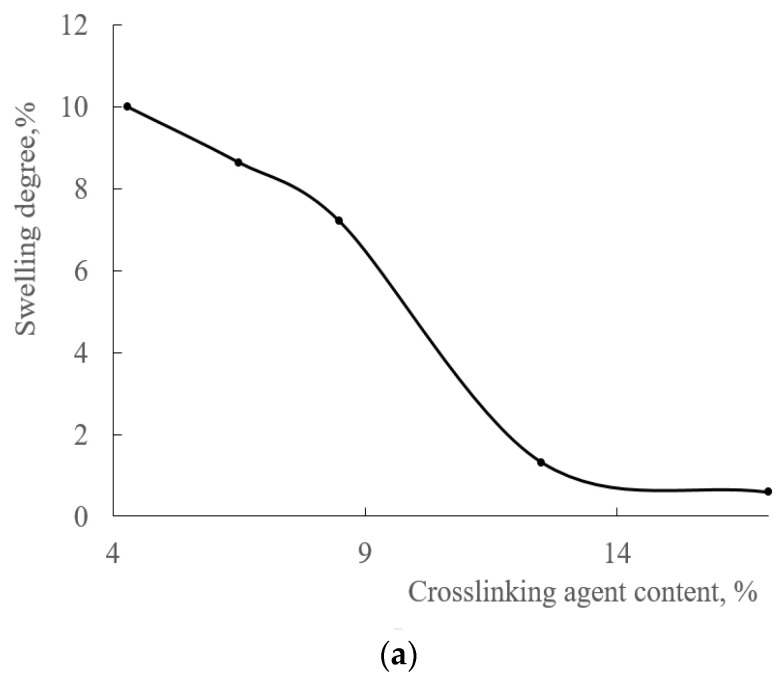

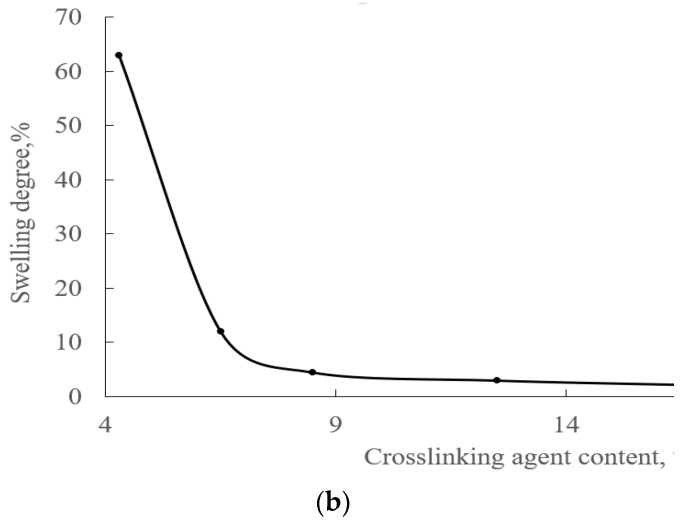
Degree of swelling of the coating as the function of hardener content (HMMM as the curing agent. PTSA was used as a catalyst in the amount of 5 wt.% of the curing agent. Terpolymer containing 45 mol.% (**a**) and 20 mol.% (**b**) of HEMA.

When HMMM is used as a crosslinking agent, the content of the mesh polymer in the coatings is approximately at the same level, regardless of the hardener and HEMA links in the copolymer. However, the density of the resulting chemical mesh is as important as the amount of mesh polymer in coating. The density of the chemical mesh was assessed by looking into the change in swelling of coatings in MPA. The swelling degree of the coatings decreases when the content of crosslinking agent in the coating increases (Figure 8). This indicates an increase in the density of chemical mesh, while the content of mesh polymer in the coating is the same. As the amount of HEMA in the copolymer decreased to 20 mol.%, the change in thickness of coatings increased from 0.6 to 2% (Figure 8b) (curing agent content 17 wt.%). This happened because the concentration of functional groups in the acrylic oligomer also decreased. Consequently, the functionality of oligomer decreased during curing, and the forming chemical mesh became less dense.

**Figure 9:**
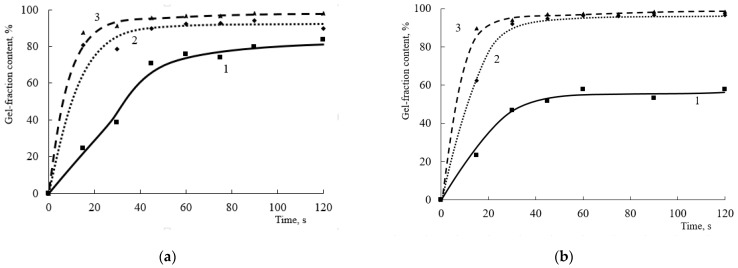

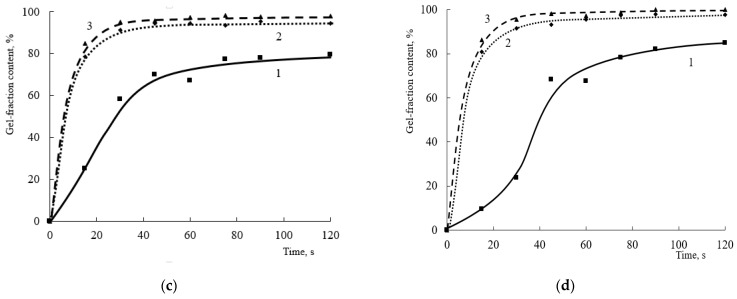
Dependence of the gel fraction content on the curing time of the compositions in the presence of TBMG (HEMA content in the terpolymer, mol.%: (**a**)—45; (**b**)—32; (**c**)—25; (**d**)—20. Catalyst content is 5 wt.%. of mass of crosslinking agent. TBMG content, wt.%: 1—4.3; 2—6.5; 3—8.5; 4—12.8; 5—17).

Changes in crosslinking agent structure and functionality can result in changes in curing speed and depth. In this work, a TBMG-based oligomer was used as the second crosslinking agent. Due to the lower functionality of this oligomer and the larger blocking group (a low-molecular-weight product based on this group would be more energy-consuming to remove from the film), its reactivity will be lower compared to that of HMMM. This is confirmed by the results obtained by studying the curing process of the compositions (Figure 9). The initial curing rate and the limiting amount of gel fraction in the composition decrease at low concentrations of crosslinking agent.

**Figure 10:**
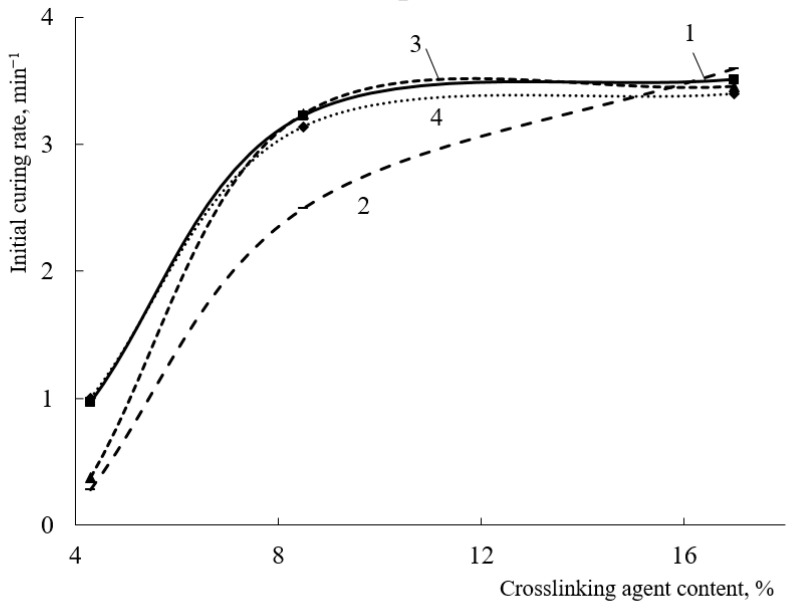
Dependence of the initial curing rate of compositions on the crosslinking agent content (TBMG-based oligomer was used as a crosslinking agent. The catalyst concentration is 5 wt.% of mass of crosslinking agent. HEMA content in the terpolymer, mol.%: 1—45; 2—32; 3—25; 4—20).

Curing with small amounts of crosslinking agent (4.3 wt.%) proceeds at significantly lower speeds (from 0.2 to 1 min^−1^ depending on the HEMA content in the copolymer), and the content of the mesh polymer does not exceed 25% (Figure 9 and Figure 10). At a similar concentration of HMMM in the composition, the initial curing rate is 2.8 to 3.4 min^−1^ (Figure 3), and the content of the mesh polymer is at 75–85% (Figure 2). As the amount of crosslinking agent increases, the initial curing rate of the composition and the content of the gel fraction increase. At a crosslinking agent concentration of 17 wt.%, the initial curing rate of the composition and the content of the gel fraction become the same regardless of the HEMA content in the composition of the copolymer (Figure 9 and Figure 10). This confirms the assumption that the crosslinking oligomer based on tetrakis(butoxymethyl)glycoluril is less reactive, but the lower reactivity in fast-curing systems gives a way to reduce internal stresses in the film as well as to reduce the number of defects in the forming film.

As the concentration of the crosslinking agent in the composition increases, both the content of the crosslinking polymer and the initial curing rate increase. This trend is observed for all the oligomers studied, regardless of the share of HEMA in the copolymer. For curing times greater than 90 s, a difference in mesh polymer content is observed at a crosslinking agent concentration of 4.3 wt.%. As the concentration of the crosslinking agent increases above 8.5 wt.%, the mesh polymer content remains at ~98% (Figure 9).

**Figure 11:**
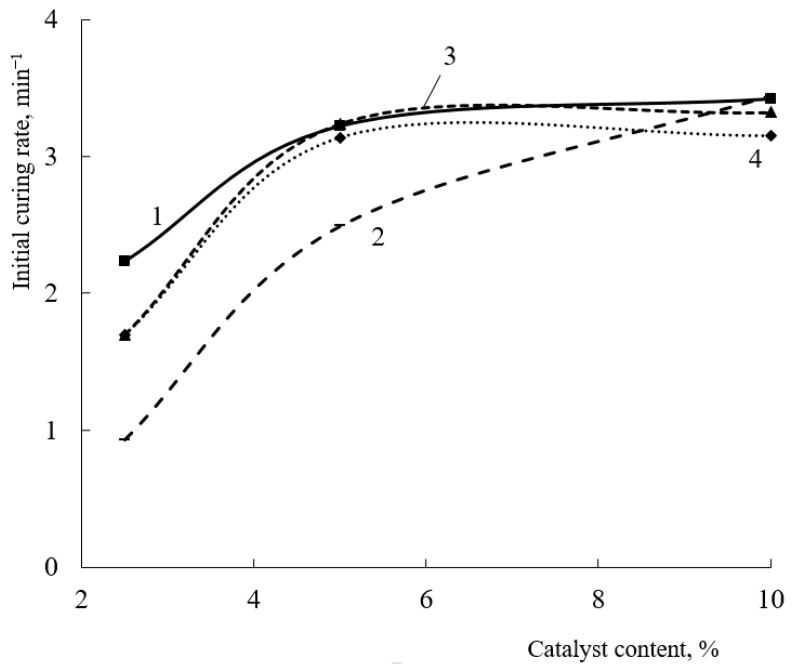
Dependence of initial curing rate of compositions on curing catalyst concentration (Crosslinking agent content—TBMG is 8.5 wt.%. HEMA content in the copolymer, mol.%: 1—45; 2—32; 3—25; 4—20).

Similar dependencies are observed when the concentration of acid catalyst is increased (Figure 11).

As the amount of PTSA in the composition increases, the content of the mesh polymer and the initial curing rate increase regardless of the proportion of HEMA in the oligomer. As a result of these studies, it was noted that the 32 mol.% HEMA-based oligomer had the lowest activity in curing in the presence of TBMG. The use of TBMG as a hardener leads to monotonic dependences, which is probably due to a smaller fraction of the side processes that happen during curing, in particular, the absence of self-condensation processes characteristic of HMMM.

**Figure 12:**
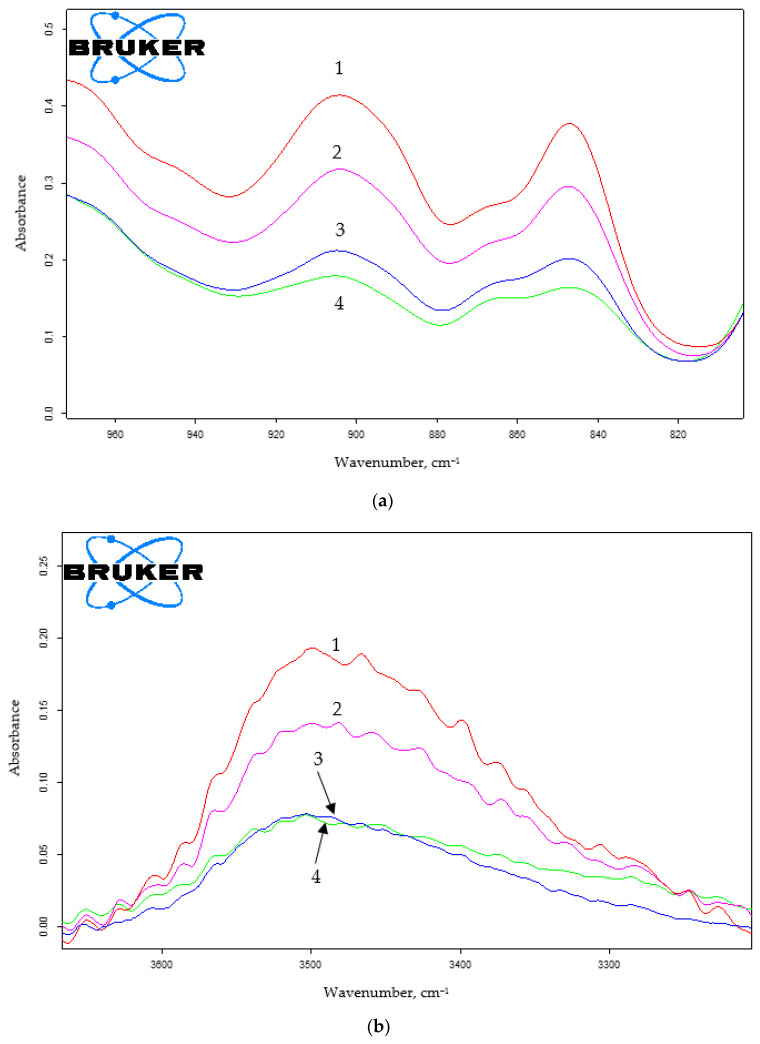
IR spectra of coatings based on terpolymer containing 45 mol.% of HEMA and TBMG as the curing agent. The area of varying the content of methoxyl (**a**) and hydroxyl (**b**) groups (PTSA was used as a catalyst in the amount of 5 wt.% of the curing agent. TBMG content, wt.%: 1—6.5; 2—8.5; 3—12.8; 4—17).

When using TBMG, we observed a decrease in the intensity of both the bands at 3500 cm^−1^, which correspond to the hydroxyl group of the oligomer, and also the band at 905 cm^−1^ that corresponds to the vibrations of the alkoxy group in the cross-linking agent (Figure 12 and Figure S3).

**Figure 13:**
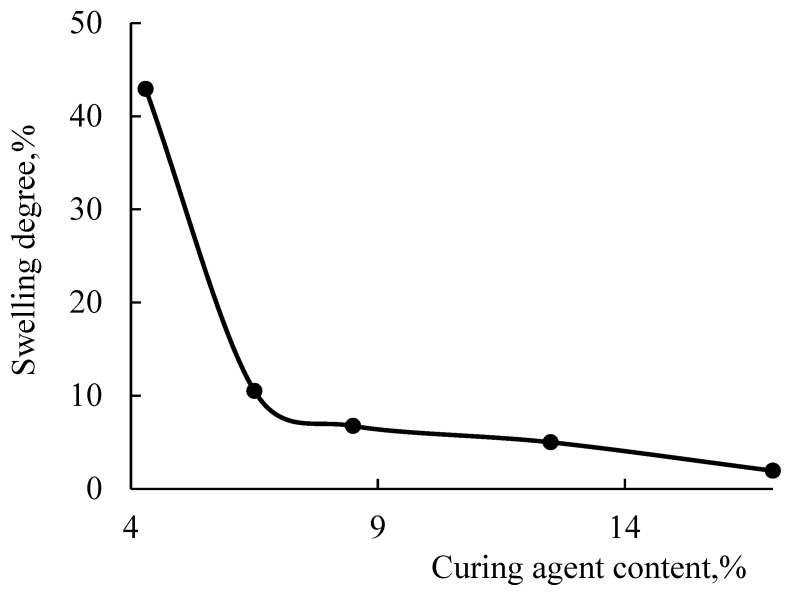
Degree of swelling of the coating as the function of hardener content (TBMG as the curing agent. PTSA was used as a catalyst in the amount of 5 wt.% of the curing agent. Terpolymer containing 45 mol.% of HEMA).

The swelling degree also decreases when using TBMG, while the content of crosslinking agent increased in the composition (Figure 13).

**Figure 14:**
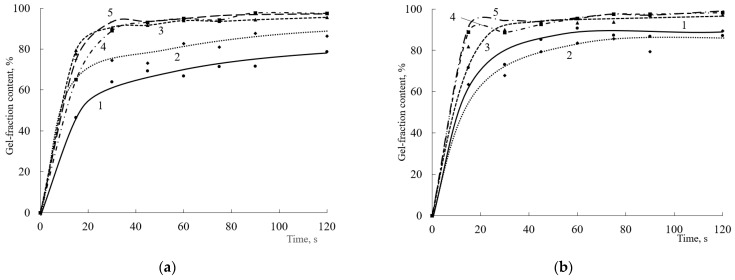

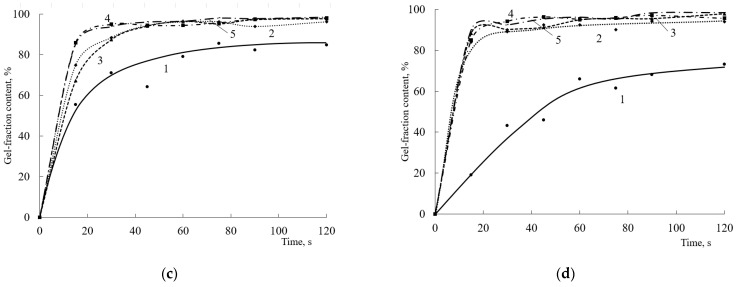
Dependence of the gel fraction content on the curing time of the compositions in the presence of TMMG (HEMA content in the terpolymer, mol.%: (**a**)—45; (**b**)—32; (**c**)—25; (**d**)—20. Catalyst content is 5 wt.% of mass of the crosslinking agent. TMMG content, wt.%: 1—4.3; 2—6.5; 3—8.5; 4—12.8; 5—17).

To assess what exactly has a greater effect on the curing process, the functionality of the crosslinking agent or the substituent blocking the functional group, the study considered the curing of the same oligomers with TMMG. Using TMMG, a crosslinking agent showed an intermediate activity between HMMM-based and TBMG-based systems (Figure 14).

This is because TMMG has a lower functionality than HMMM, which reduces the concentration of reactive groups if the amount of hardener in the system is the same. This reduces the curing rate of the composition. Compared to TBMG, the substituent that blocks the reactive group in a TMMG molecule takes less space, which facilitates its easier and faster removal.

**Figure 15:**
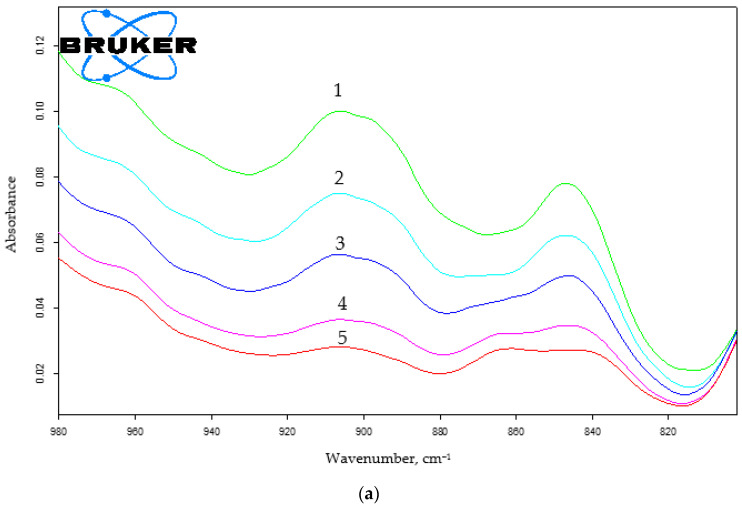

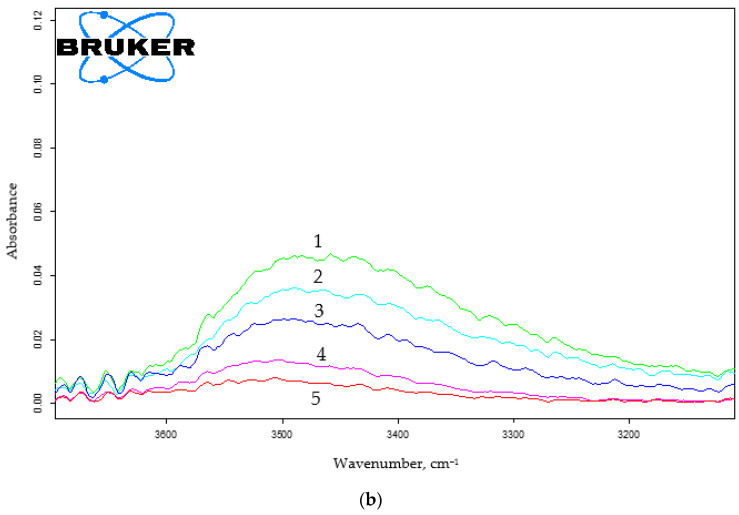
IR spectra of coatings based on terpolymer containing 45 mol.% of HEMA and TMMG as the curing agent. The area of varying the content of methoxyl (**a**) and hydroxyl (**b**) groups (PTSA was used as a catalyst in the amount of 5 wt.% of the curing agent. TMMG content, wt.%: 1—4.3; 2—6.5; 3—8.5; 4—12.8; 5—17).

Using TMMG as a crosslinking agent, we found the similar shift in the functional group content as with HMMM- and TBMG-based compositions (Figure 15 and Figure S4).

**Figure 16:**
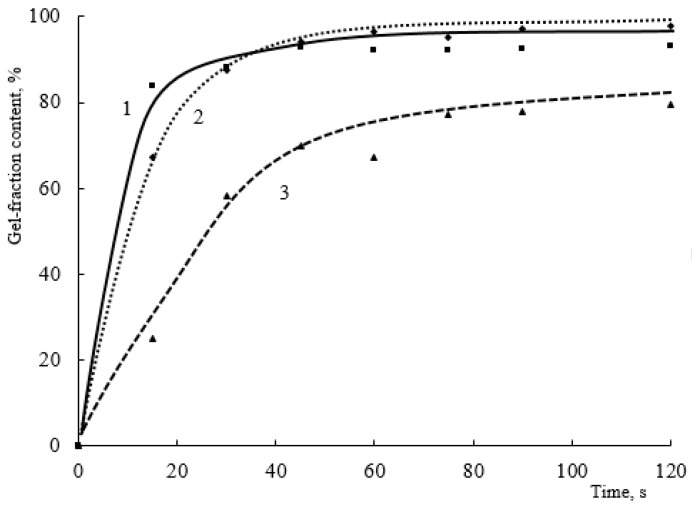
Dependence of the gel fraction content on the curing time of the compositions in the presence of different crosslinking agents (HEMA content in the terpolymer is 20 mol.%. Crosslinking agent concentration is 4.3 wt.%. Catalyst content is 5 wt.%. of mass of the crosslinking agent. Crosslinking agent: 1—HMMM; 2—TMMG; 3—TBMG).

The most obvious differences in crosslinking agent activity can be seen in compositions using copolymers with a low content of hydroxyl groups and a small amount of hardener (Figure 16).

It can be seen that the greater functionality of HMMM leads to a higher initial curing rate of the oligomers, while the limit content of the gel fraction for both HMMM and TMMG is at the same level. Reducing the functionality of the hardener and replacing the methoxyl groups with butoxyl groups, as in the case of TBMG, lead to both a significant decrease in the initial curing rate and a decrease in the ultimate gel fraction content in the coatings.

When comparing the IR spectra of coatings obtained using different hardeners, it was also evident that, regarding the attainable hydroxyl group reaction conversion, crosslinkers can be arranged in the following order: HMMM > TMMG > TBMG (Figure S5). This is in agreement with sol-gel analysis results. It should be noted that the methoxyl group content in HMMM- and TMMH-based coatings remains the same. The greater possible conversion of hydroxyl group reaction and simultaneously the same content of methoxyl groups when using HMMM is due to its greater functionality and, consequently, a higher initial concentration of —OCH_3_ groups.

As can be seen from the presented curves, the lowest change in thickness is achieved when using HMMM as a crosslinking agent (0.6% at 17 wt.%), which is due to the higher functionality of the hardener (Table 3). Among glycoluril derivatives, the lowest change in thickness is observed with TMMG (1% at 17 wt.% of crosslinking agent, versus 2% with TBMG), since this hardener is more reactive. This is due to less steric hindrance during curing because of the smaller volume of blocking group.

## 4. Conclusions

The use of HMMM as a crosslinking agent leads to a 200% increase in the initial curing rate of the composition as compared to TBMG. The content of the mesh polymer in the initial moments of curing is also significantly higher in the compositions cured with HMMM. In the presence of HMMM, the resulting coating is less compositionally uniform because HMMM is prone to a self-condensation reaction in the presence of a strong acid such as PTSA. Using TBMG as a crosslinking agent reduces the initial curing rate and the content of the crosslinking polymer at the beginning of the curing process, but the resulting coatings will be less defective due to a more uniform crosslinking process. The concentration of the crosslinking agent and the acid catalyst significantly affects the curing process when the proportion of HEMA in the copolymer is 20 mol.% or less. With large amounts of it, this effect is insignificant with curing time over 90 s.

## Figures and Tables

**Table 1:** Compositions and molecular weight properties of copolymers.

Sample	Composition of Copolymer, mol.%			Molecular Weight Properties		
	Methyl Methacrylate	Styrene	2-Hydroxyethyl Methacrylate	M_n_	M_w_	P_d_
1	18	37	45	7900	19,600	2.5
2	31	37	32	9400	21,250	2.3
3	38	37	25	9300	20,200	2.2
4	43	37	20	10,100	22,600	2.2

**Table 2:** Three-dimensional solubility parameter and dynamic viscosity of used solvents.

Solvent	Viscosity, mPa∙s	Solubility Parameters			
		Total Solubility Parameter (δ)	Dispersion Component (δ_d_)	Polar Component (δ_p_)	The Component Responsible for Hydrogen Bonds (δ_H_)
THF	0.6	18.5	16.8	5.7	8.0
MOP	1.9	21.9	15.6	7.2	13.6
MPA	1.1	18.4	16.1	6.6	6.6

**Table 3:** Swelling degree of coatings.

Sample	HEMA Content in Terpolymer, mol.%	Curing Agent	Curing Agent Content, wt.%	Swelling Degree, %
1	45	HMMM	17	0.58
2		TBMG	17	1.95
3		TMMG	17	0.98

## Data Availability

The data presented in this study are available on request from the corresponding author.

## Supplementary Materials

The following supporting information can be downloaded at: https://www.mdpi.com/article/10.3390/polym15092187/s1, Figure S1: IR spectra of coatings based on terpolymer containing 45 mol.% of HEMA and HMMM as the curing agent (PTSA was used as a catalyst in the amount of 5 wt.% of the curing agent. HMMM content, wt.%: 1—4.3; 2—6.5; 3—8.5; 4—12.8; 5—17); Figure S2: IR spectra of coatings based on terpolymer containing 20 mol.% of HEMA and HMMM as the curing agent (PTSA was used as a catalyst in the amount of 5 wt.% of the curing agent. HMMM content, wt.%: 1—4.3; 2—6.5; 3—8.5; 4—12.8; 5—17); Figure S3: IR spectra of coatings based on terpolymer containing 20 mol.% of HEMA and TBMG as the curing agent (PTSA was used as a catalyst in the amount of 5 wt.% of the curing agent. TBMG content, wt.%: 1—6.5; 2—8.5; 3—12.8; 4—17); Figure S4: IR spectra of coatings based on terpolymer containing 45 mol.% of HEMA and TMMG as the curing agent (PTSA was used as a catalyst in the amount of 5 wt.% of the curing agent. TMMG content, wt.%: 1—4.3; 2—6.5; 3—8.5; 4—12.8; 5—17); Figure S5: IR spectra of coatings based on terpolymer containing 45 mol.% of HEMA and different curing agents (PTSA was used as a catalyst in the amount of 5 wt.% of the curing agent. Curing agent: 1—TBMG; 2—TMMG; 3—HMMM).
