# Supplementary material for: Curing of Poly(styrene-co-methyl methacrylate-co-2-hydroxyethyl methacrylate) Terpolymers in the Presence of Amino Compounds of Different Structures
Source: Polymers (Basel). 2023 May 4;15(9):2187. doi: 10.3390/polym15092187 (PMC10180959; doi:10.3390/polym15092187)
Supplement: Supplementary file 1 [file polymers-15-02187-s001.zip › polymers-2327542-supplementary.pdf]

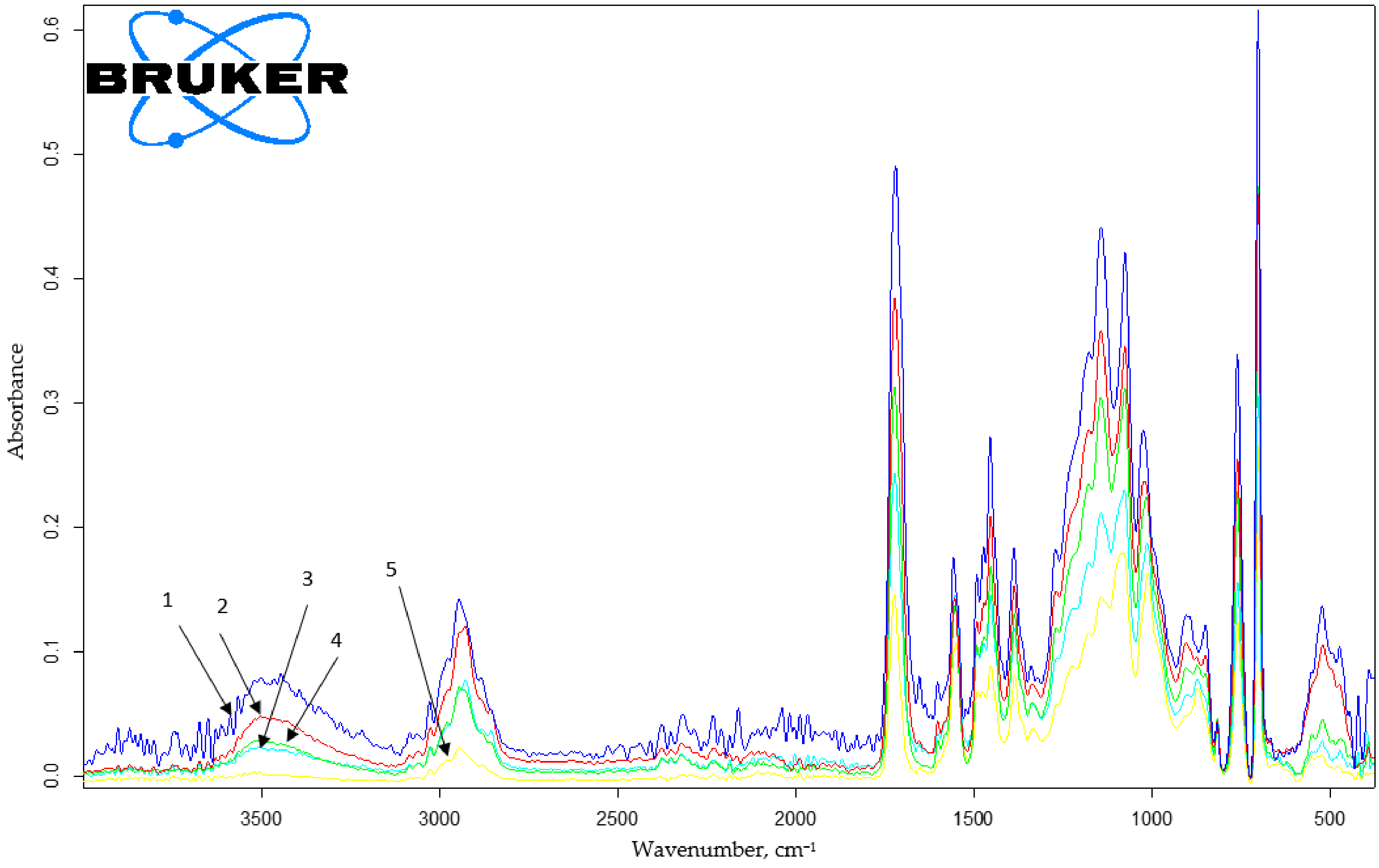

Figure S1. IR spectra of coatings based on terpolymer containing 45 mol.% of HEMA and HMMM as the curing agent (PTSA was used as a catalyst in the amount of 5 wt.% of the curing agent. HMMM content, wt.%: 1 – 4.3; 2 - 6.5; 3 - 8.5; 4 - 12.8; 5 – 17)

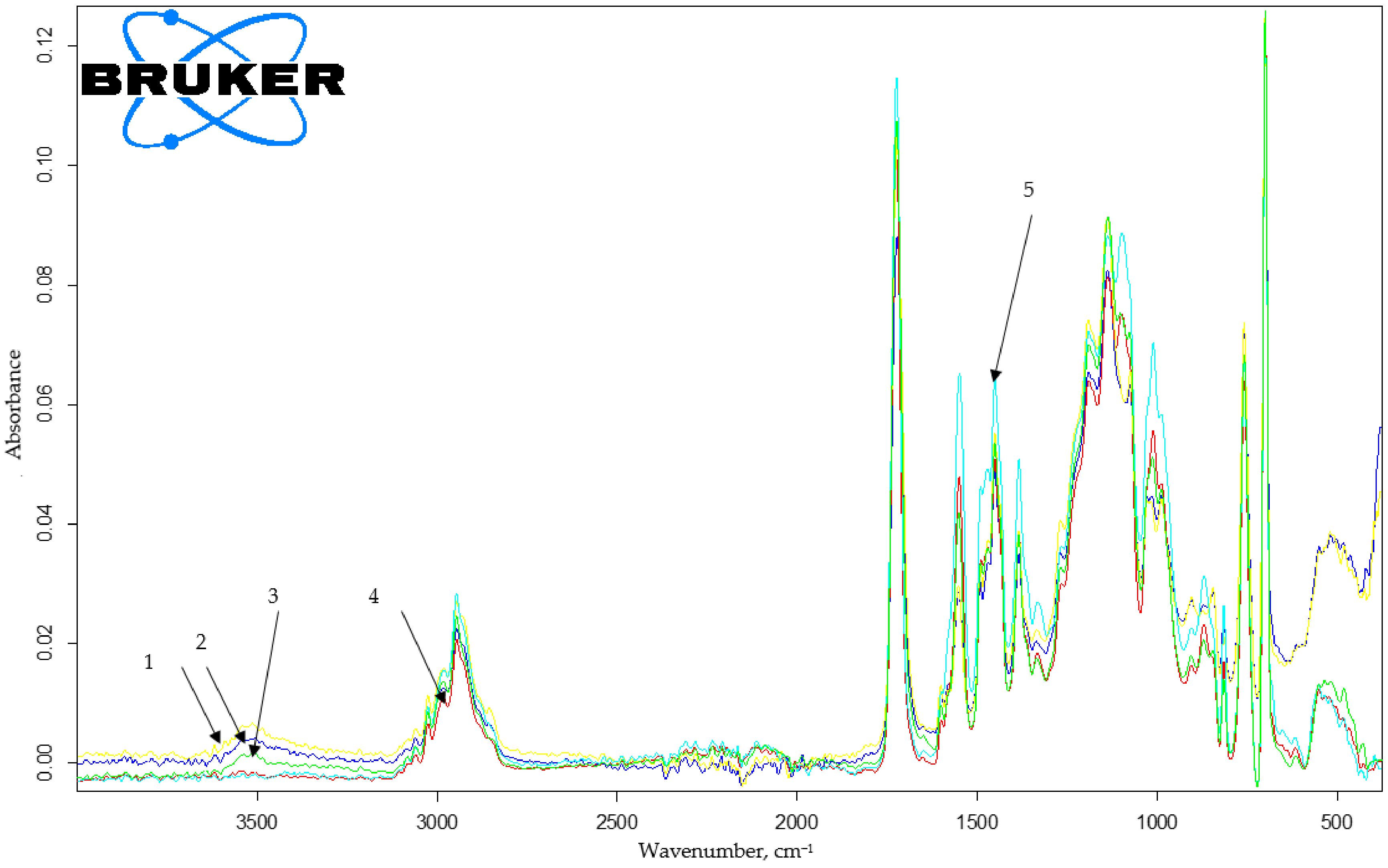

Figure S2. IR spectra of coatings based on terpolymer containing 20 mol.% of HEMA and HMMM as the curing agent (PTSA was used as a catalyst in the amount of 5 wt.% of the curing agent. HMMM content, wt.%: 1 – 4.3; 2 - 6.5; 3 - 8.5; 4 - 12.8; 5 – 17)

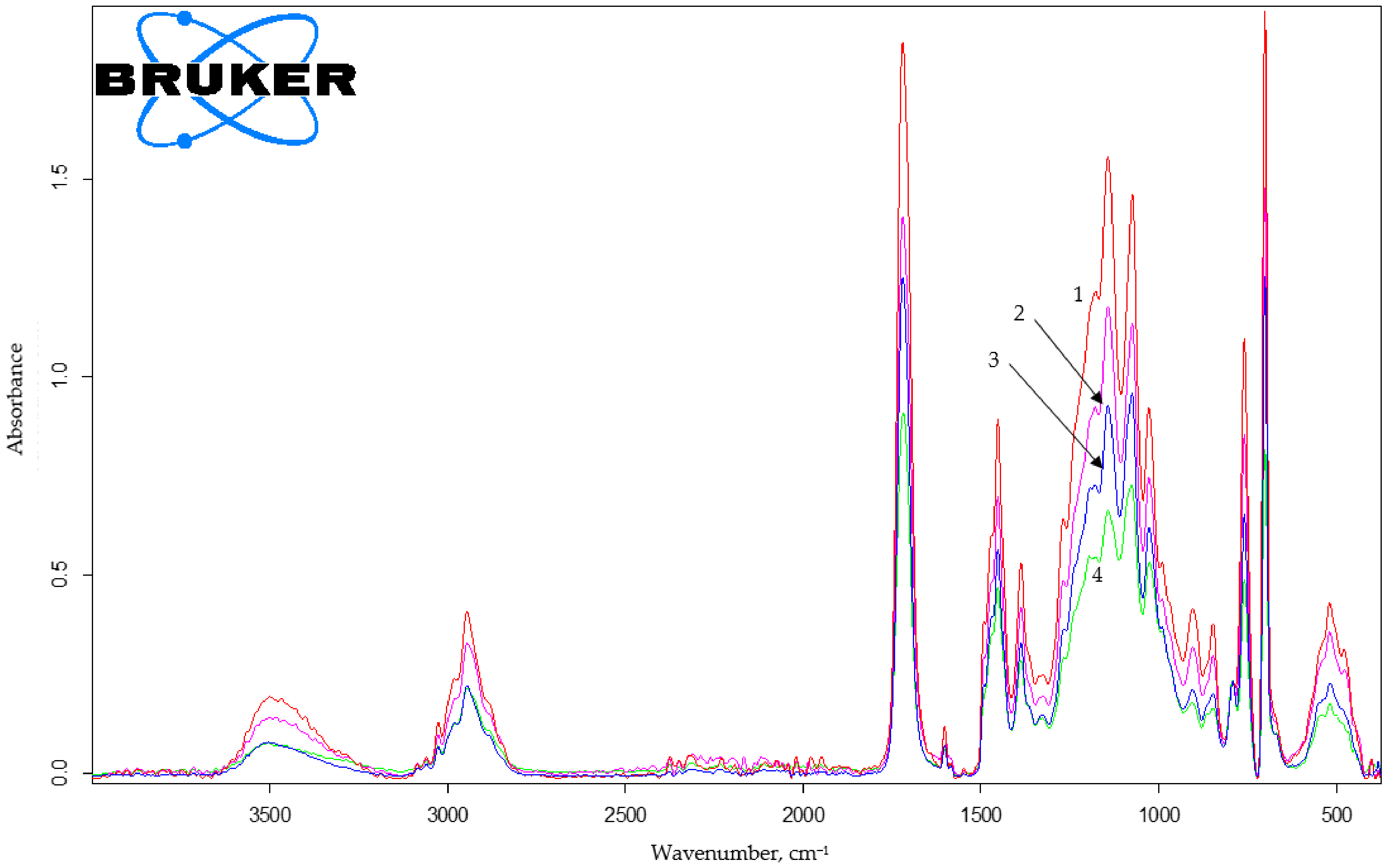

Figure S3. IR spectra of coatings based on terpolymer containing 20 mol.% of HEMA and TBMG as the curing agent (PTSA was used as a catalyst in the amount of 5 wt.% of the curing agent. TBMG content, wt.%: 1 – 6.5; 2 – 8.5; 3 – 12.8; 4 - 17)

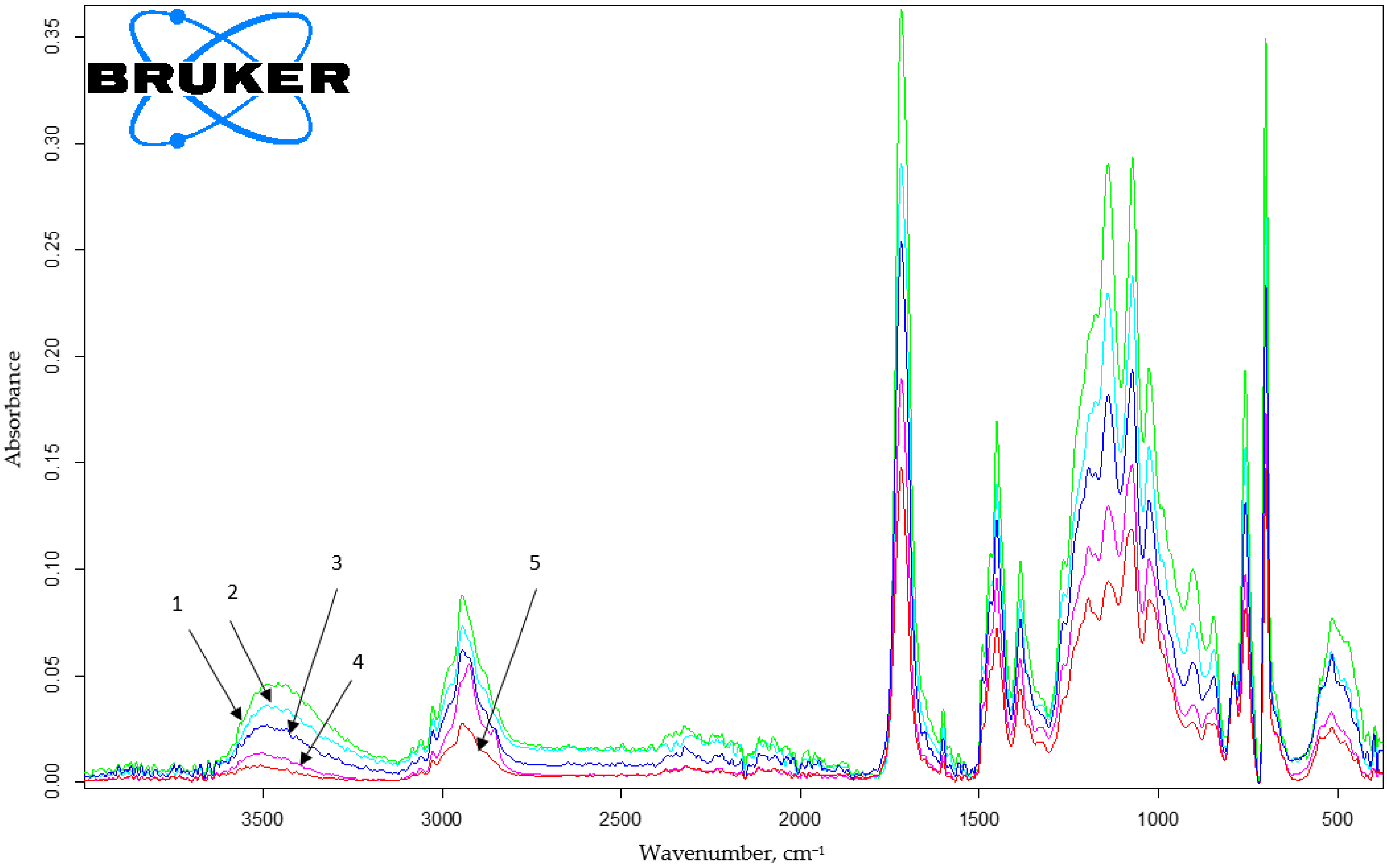

Figure S4. IR spectra of coatings based on terpolymer containing 45 mol.% of HEMA and TMMG as the curing agent (PTSA was used as a catalyst in the amount of 5 wt.% of the curing agent. TMMG content, wt.%: 1 – 4.3; 2 - 6.5; 3 - 8.5; 4 - 12.8; 5 – 17)

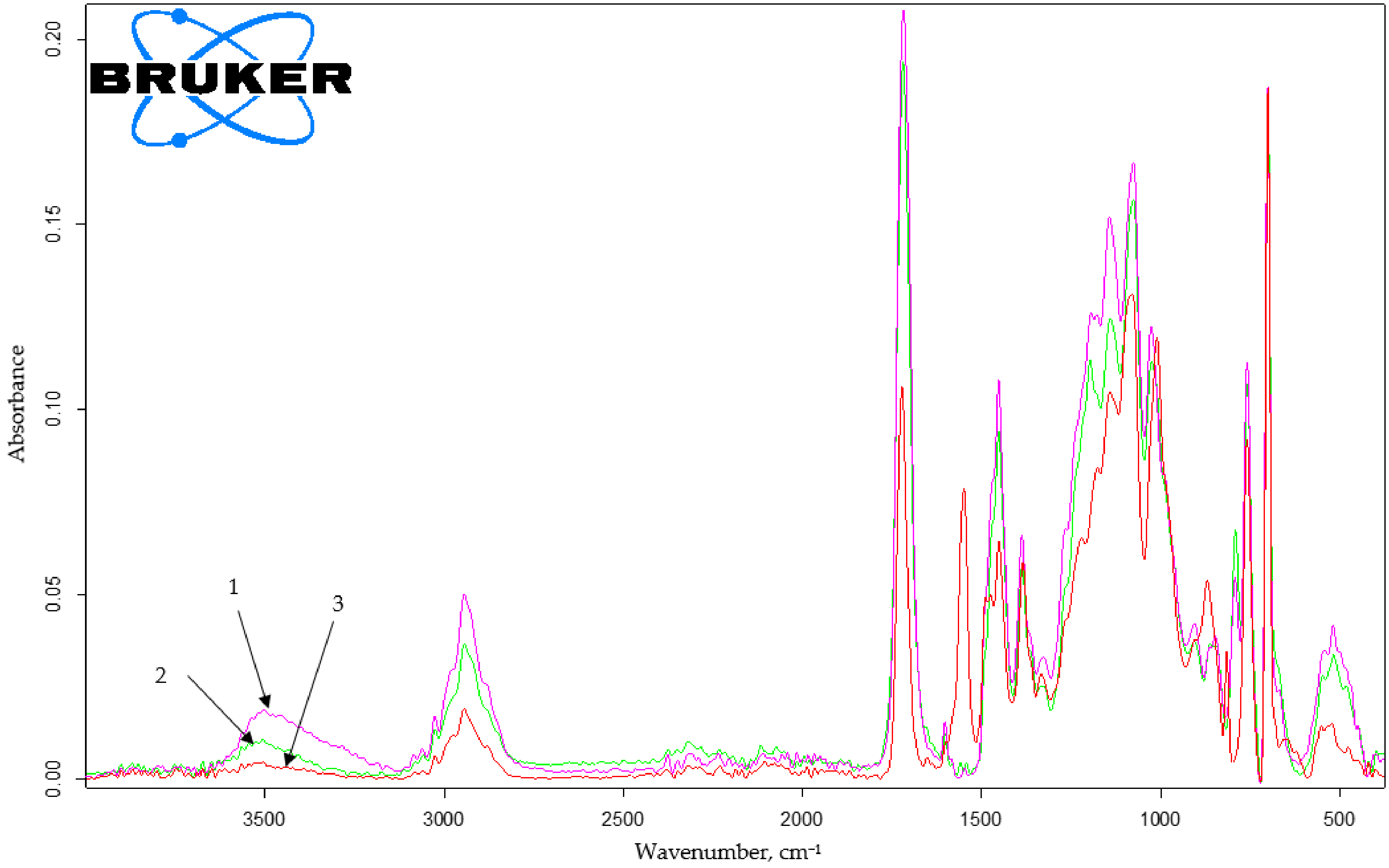

Figure S5. IR spectra of coatings based on terpolymer containing 45 mol.% of HEMA and different curing agents (PTSA was used as a catalyst in the amount of 5 wt.% of the curing agent. Curing agent: 1 – TBMG; 2 –TMMG; 3 – HMMM)
